# Establishment and characterization of two 5-fluorouracil-resistant hepatocellular carcinoma cell lines

**DOI:** 10.3892/ijo.2011.1300

**Published:** 2011-12-15

**Authors:** KAZUYA UCHIBORI, ATSUSHI KASAMATSU, MASAHIKO SUNAGA, SATOSHI YOKOTA, TOMOYA SAKURADA, ERIKO KOBAYASHI, MASAHARU YOSHIKAWA, KATSUHIRO UZAWA, SHIRO UEDA, HIDEKI TANZAWA, NOBUNORI SATO

**Affiliations:** 1 Department of Clinical Education and Research, Graduate School of Pharmaceutical Sciences, Chiba University, 1-8-1 Inohana, Chuo-ku, Chiba 260-8670; 2 Department of Medicine and Clinical Oncology, Graduate School of Medicine, Chiba University, 1-8-1 Inohana, Chuo-ku, Chiba 260-8670; 3 Department of Clinical Molecular Biology, Graduate School of Medicine, Chiba University, 1-8-1 Inohana, Chuo-ku, Chiba 260-8670; 4 Division of Oral-Maxillofacial Surgery, Chiba University Hospital, 1-8-1 Inohana, Chuo-ku, Chiba 260-8670; 5 Chiba Central Medical Center, 1835-1 Kasori-cho, Wakaba-ku, Chiba 264-0017, Japan

**Keywords:** 5-fluorouracil, drug resistance, hepatocellular carcinoma, epithelial-to-mesenchymal transition

## Abstract

5-Fluorouracil (5-FU) chemotherapy is the first choice treatment for advanced hepatocellular carcinoma (HCC), and resistance is the major obstacle to successful treatment. Recent studies have reported that epithelial-to-mesenchymal transition (EMT) is associated with chemoresistance in cancers. We speculated that EMT and 5-FU metabolism are related to the mechanism of 5-FU resistance. First, two 5-FU-resistant cell lines, HLF-R4 and HLF-R10, were established from the HLF undifferentiated human HCC cell line. Whereas cell growth was similar in the HLF and HLF-R cell lines, HLF-Rs are about 4- and 10-fold more resistant compared with the HLF cells; thus, we named these cell lines HLF-R4 and HLF-R10, respectively. The terminal deoxyribonucleotidyl transferase-mediated dUTP nick end labeling assay also showed a dramatically decreased number of apoptotic cells in the HLF-Rs after treatment with 5-FU. We next assessed the characteristics of the HLF, HLF-R4 and HLF-R10 cells. Consistent with our hypothesis, the HLF-Rs had typical morphologic phenotypes of EMT, loss of cell-cell adhesion, spindle-shaped morphology and increased formation of pseudopodia. Real-time quantitative reverse transcriptase polymerase chain reaction data showed downregulated *E-cadherin* and upregulated *Twist-1* and also indicated that EMT changes occurred in the HLF-Rs. We also found decreased *ribonucleotide reductase* and increased *multidrug resistance protein 5* genes in the HLF-R cells. Our results suggested that the metabolism of EMT and 5-FU has important roles in 5-FU chemoresistance in the HLF-R cells, and that the HLF-R cells would be useful *in vitro* models for understanding the 5-FU-resistant mechanisms in HCC.

## Introduction

Hepatocellular carcinoma (HCC) is one of the most frequently occurring malignant tumors and a common cause of cancer mortality worldwide (1). The results of surgical treatments for advanced HCC, such as liver resection or liver transplantation, have not been satisfactory. To date, chemotherapy, including transcatheter arterial infusion, is the first choice for advanced HCC (2,3). 5-Fluorouracil (5-FU) is a commonly used chemotherapeutic agent that is effective for treating a wide variety of malignant tumors. However, the effectiveness of the chemotherapy is limited because of acquired or intrinsic drug resistance (4,5).

The development of resistance to 5-FU appears to be a major impediment to the successful chemotherapy of human cancers. 5-FU decreases the biosynthesis of pyrimidine nucleotides by inhibiting thymidylate synthase, which catalyzes the rate-limiting step in DNA synthesis (6–10). Although the mechanism of resistance to 5-FU remains unclear, a possible mechanism is that alterations of plasma membrane proteins reduce the accumulation of 5-FU within tumor cells (11). Two studies also reported that epithelial-to-mesenchymal transition (EMT) was closely related to chemoresistance in the colorectal and pancreatic cancers (12,13).

EMT is initially observed in embryonic development in which cells lose epithelial characteristics and gain mesenchymal properties to increase motility and invasiveness (12,14). Previous research has suggested that EMT is also important in tumor progression, metastasis, and chemoresistance (14,15) and is induced by growth factors, such as hepatocyte growth factor, transforming growth factor β, and epidermal growth factor, implicated in these processes (16).

Tissue culture systems have been established to study the biochemical, physiologic, and genetic bases of alterations that result in development of multidrug resistance. To understand drug resistance well, establishing cultured cell lines resistant to anticancer drugs is necessary. In the current study, we established two 5-FU-resistant cell lines, HLF-R4 and HLF-R10, from an HCC cell line and investigated for the first time the mechanisms of 5-FU resistance including EMT.

## Materials and methods

### Cell lines

The HLF cell line, derived from undifferentiated human hepatocellular carcinoma, was obtained from the Japanese Cancer Research Resources Bank and maintained in Dulbecco's modified Eagle's medium (DMEM) (Gibco, Carlsbad, CA) supplemented with 10% fetal bovine serum (FBS) (Gibco) and 100 units/ml penicillin and streptomycin (Gibco). According to previously described methods (17–20), two 5-FU-resistant sublines, HLF-Rs, were established by repeated subcultures in the presence of stepwise-increases in concentrations of 5-FU (5, 7.5, 10 and 20 μM) (Wako, Osaka, Japan). Because the chemoresistance of both cell lines are 4- and 10-fold increased, respectively, the sublines were named HLF-R4 and HLF-R10, and they are fully resistant to 5-FU and can grow exponentially in the presence of 10 and 20 μM of 5-FU. The HLF-Rs showed no loss of resistance even after 2 months of culture in a drug-free medium.

### Cell growth

To investigate cell growth in the HLF, HLF-R4, and HLF-R10 cells, we performed a cell proliferation assay. The experiments were carried out for 168 h, and the number of cells were counted every 24 h. The cells in three samples were trypsinized and counted using a hemocytometer at the indicated time points.

### Chemosensitivity assay

The cells were seeded at a concentration of 2.0×10^3^ in each well of a 96-well plate in DMEM containing 10% FBS. After 24 h, culture medium was replaced with DMEM with 10% FBS and various concentrations of 5-FU (6.25, 12.5, 25, 50, 100, 200, 400, 800 and 1600 μM). After further incubation for 72 h, a cell viability assay was carried out using the Cell Counting kit-8 according to the manufacturer's instructions (Dojindo, Kumamoto, Japan). Six wells were used for each drug concentration and the experiment was replicated three times. The 50% inhibitory concentration (IC_50_) was calculated from the survival curves.

### TUNEL assay

The cells were seeded at a concentration of 5.0×10^5^ on Lab-TekII chamber slides (Nalge Nunc International, Rochester, NY) in DMEM containing 10% FBS. After incubation for 24 h, the culture medium was replaced with DMEM with 10% FBS and 70 μM of 5-FU. After further incubation for 24 h, the cells on the chamber slides were washed twice with phosphate buffered saline, air dried, and fixed in 4% paraformaldehyde at room temperature for 30 min. The terminal deoxyribonucleotidyl transferase-mediated dUTP nick end labeling (TUNEL) assay was performed using an *In Situ* Apoptosis Detection kit according to the manufacturer's instructions (Takara, Tokyo, Japan). The cells were viewed and photographed under a fluorescence microscope.

### Preparation of cDNA

Total RNA was isolated using an RNeasy Mini kit (Qiagen, Chatsworth, CA) according to the manufacturer's instructions. cDNA was generated from 5 μg of total RNA using an Ominiscript RT kit (Qiagen) and random hexamer (Sigma Genosys, Ishikari, Japan).

### mRNA expression analysis

Real-time quantitative reverse transcriptase polymerase chain reaction (qRT-PCR) was performed to evaluate the expression levels of five target genes in HLF and its derivatives. qRT-PCR was carried out with one method using a LightCycler FastStart DNA Master SYBR Green 1 kit (Roche, Mannheim, Germany). The specific primers for the target genes are listed in Table I. Amplified products were analyzed by 3% agarose gel electrophoresis to ascertain size and purity. The PCR reactions using the LightCycler apparatus were performed in a final volume of 20 μl of a reaction mixture comprised of 2 μl of FirstStart DNA Master SYBR Green I mix, 3 mM MgCl_2_, and l μM of the primers, according to the manufacturer's instructions. The reaction mixture was loaded into glass capillary tubes and subjected to an initial denaturation at 95°C for 10 min, followed by 45 rounds of amplification at 95°C (10 sec) for denaturation, 62°C (10 sec) for annealing, and 72°C (10 sec) for extension, with a temperature slope of 20°C/sec. The transcript amounts for the target genes were estimated from the respective standard curves and normalized to the *glyceraldehyde-3-phosphate dehydrogenase (GAPDH)* transcript amount determined in corresponding samples.

### Statistical analysis

The statistical significance was evaluated using the Mann-Whitney U test. P<0.05 was considered statistically significant.

## Results

### Cell growth

**Figure 1:**
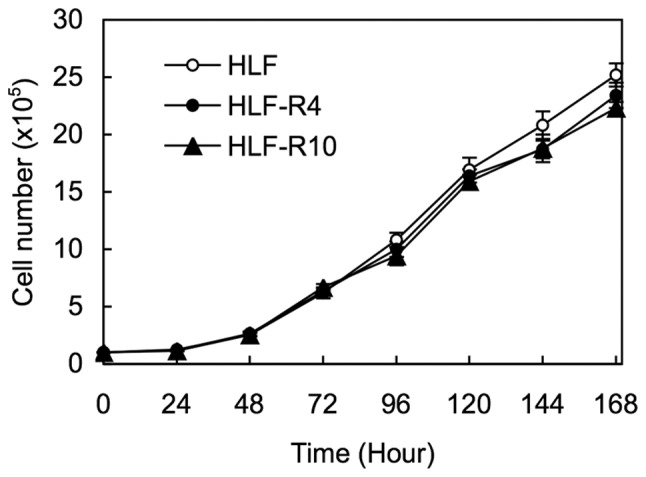
Cell growth assay. The experiments were carried out for 168 h, with counting of the number of cells every 24 h. The proliferation rates of the HLF-R4 (●) and HLF-R10 (▴) cells are similar to that of the HLF cells (○). The results are expressed as the means ± standard error of the mean values from three assays.

To obtain 5-FU-resistant cells, the HLF cells were treated with increasing concentrations of 5-FU up to 20 μM, and two clones, designated HLF-R4 and HLF-R10, were established almost 1.5 years later. There were no significant differences in cellular proliferation among the HLF, HLF-R4, and HLF-R10 cells (Fig. 1).

### Chemosensitivity assay

**Figure 2:**
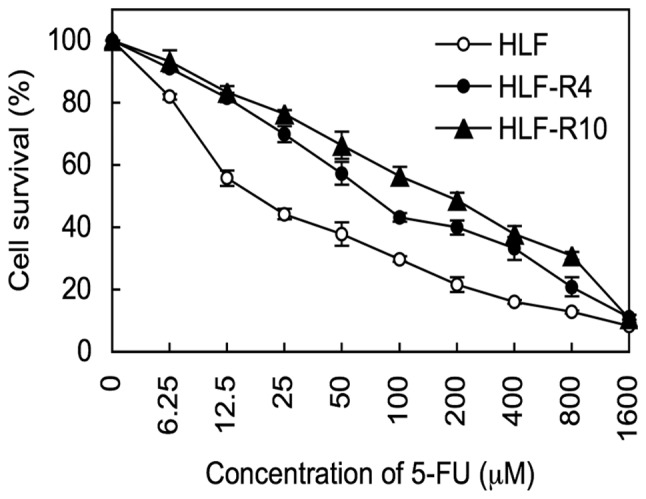
Chemosensitivity assay. IC_50_ values of 5-FU for the HLF (○), HLF-R4 (●), and HLF-R10 (▴) cells are 17.92 μM, 69.80 μM and 193.47 μM, respectively. Therefore, the 5-FU resistance of the HLF-R4 cells is 3.9-fold, and that of the HLF-R10 cells is 10.8-fold greater than that of the HLF cells. The results are expressed as the means ± standard error of the mean values from three assays.

The IC_50_ data indicated that the 5-FU-resistance levels of the HLF-R4 cells (IC_50_, 69.80 μM) and HLF-R10 cells (IC_50_, 193.47 μM) were 3.9- and 10.8-fold greater than that of the HLF cells (IC_50_, 17.92 μM) (Fig. 2).

### TUNEL assay

**Figure 3:**
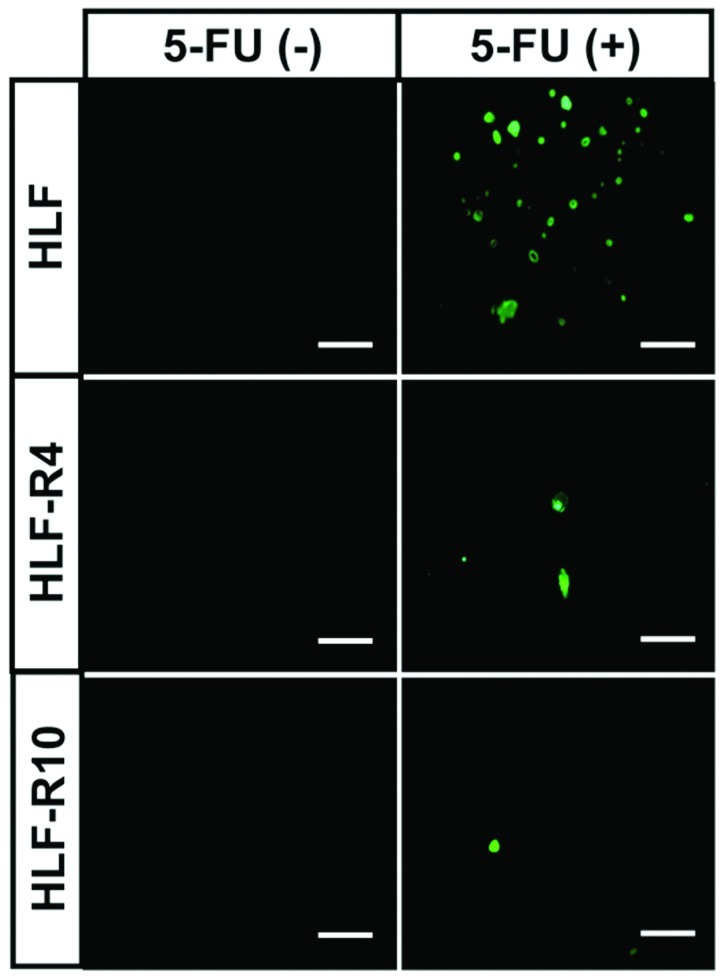
TUNEL assay. To evaluate the effect of 5-FU on the HLF, HLF-R4, and HLF-R10 cells, a TUNEL assay was used to confirm the apoptotic changes. TUNEL staining shows a dramatic increase in the number of cells that were stained green, which indicates that apoptosis occurred in HLF cells exposed to 5-FU. 5-FU-induced apoptosis is inhibited in the HLF-R4 and HLF-R10 cells. The experiments were repeated three times with similar results. Scale bar = 50 μm.

TUNEL staining showed a dramatic increase in the number of cells that were stained green, which indicated that apoptosis occurred in HLF cells exposed to 5-FU, whereas 5-FU-induced apoptosis was prevented in the HLF-R4 and HLF-R10 cells (Fig. 3).

### Cell morphology

**Figure 4:**
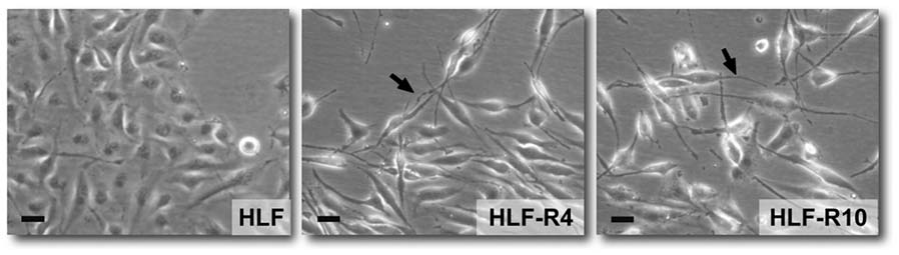
Cell morphology. 5-FU-resistant cells (HLF-R4 and HLF-R10) are morphologically distinct from their parental cell line, HLF. The resistant cells have loss of cell-cell adhesion, spindle-shaped morphology, and increased formation of pseudopodia (arrow). Scale bar, 20 μm.

The 5-FU-resistant cells, HLF-R4 and HLF-R10, were morphologically distinct from their parental cell line (Fig. 4). The resistant cells had loss of cell-cell adhesion, spindle-shaped morphology, and increased formation of pseudopodia. These changes are typical phenotypes of EMT.

### Evaluation of the expression of genes putatively related to 5-FU resistance and EMT

**Figure 5:**
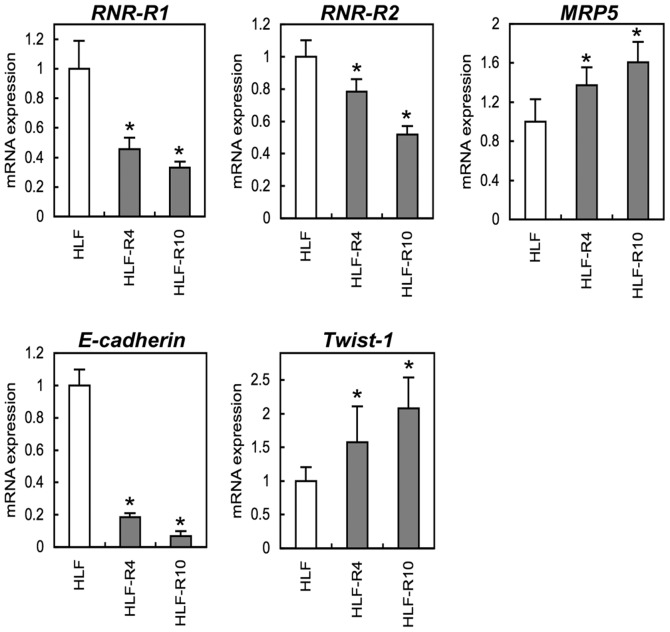
mRNA expression analysis. qRT-PCR was performed to investigate mRNA levels of *RNR-R1*, *RNR-R2*, *MRP5*, *E-cadherin*, and *Twist-1*. PCR shows up-regulation of *MRP5* and *Twist-1* and down-regulation of *RNR-R1*, *RNR-R2*, and *E-cadherin* dependent on the level of 5-FU chemoresistance. Data are expressed as the means ± standard error of the mean values from three assays (^*^p<0.05 by the Mann-Whitney U test).

Consistent with our hypothesis that EMT occurs in 5-FU-resistant cells, qRT-PCR showed decreased *E-cadherin* and increased *Twist-1* by in the HLF-Rs. Among the genes associated with 5-FU metabolism, we also found that mRNA expression of *ribonucleotide reductases* (*RNR-R1*, *RNR-R2*) was down-regulated and *multi-drug resistance protein 5 (MRP5)* was up-regulated in the HLF-Rs cells in a 5-FU-chemoresistant level-dependent manner (Fig. 5) (p<0.05).

## Discussion

5-FU is key anticancer chemotherapy used to treat solid tumors, such as gastric, colorectal, pancreas, breast, and lung carcinomas (21–25). 5-FU also has been preferentially used alone or combined with other chemotherapeutic drugs for HCCs (2,3). Although several mechanisms of 5-FU resistance have been investigated, no one mechanism completely explains the clinical response to 5-FU chemotherapy. Since it is extremely important to understand the resistance mechanism in order to develop better treatments, establishing 5-FU-resistant cells was indispensable to this type of study. Cell lines that are resistant to 5-FU have been established from several cancers (6,26–28). However, to date, only the Bel7402/5-FU cell line from HCC has been reported to be resistant to 5-FU (29). To obtain further detailed information on 5-FU resistance in HCC, in the current study we evaluated two new 5-FU-resistant cell lines, HLF-R4 and HLF-R10, from an undifferentiated HCC cell line that showed different resistance levels to 5-FU.

Consistent with previous reports that showed that 5-FU metabolism and activity of 5-FU transport were closely related to 5-FU resistance (30,31), the current data showed down-regulated *RNRs* and up-regulated *MRP5* genes. RNR, a key enzyme in 5-FU metabolism, catalyzed 5-fluorouridine diphosphate to 5-fluorodeoxyuridine diphosphate, the main precursor of 5-fluorodeoxyuridine monophosphate (FdUMP) (32). Decreased RNR activity leads to a low FdUMP level followed by inhibited thymidylate synthase cellular activity, indicating that acquired resistance to 5-FU occurs in the cells (6–10). MRP5, an energy-dependent ATP-binding cassette transporter protein (33), mediates the ATP-dependent transport of various substrates, such as monophosphate metabolites (30), across biologic membranes (34) and is responsible for broad-spectrum resistance to chemotherapy including 5-FU. 5-FU metabolism may contribute to a basic understanding of the molecular mechanisms of chemoresistance. Further study is necessary to identify other mechanisms.

In addition to typical morphologic phenotypes of EMT in the HLF-Rs cells, we found decreased E-cadherin expression caused by Twist-1 expression, which is consistent with the results of Matsuo *et al* (35). These findings reflected an important process by which cancer cells may potentially become chemoresistant. Even though the relationship between EMT and chemoresistance remains unclear, induction of EMT in 5-FU-chemoresistant HCC cells might represent a new potentially exciting research area into 5-FU-resistance mechanisms. Thus, EMT is likely to be a potential therapeutic target for development of anticancer drugs in HCCs. Our data should eventually benefit patients with advanced HCC or 5-FU-chemoresistant HCCs for whom there currently are few effective treatment options.

In conclusion, we found that alterations of enzymes affecting 5-FU transport and metabolism as well as EMT were observed in the HLF-Rs. Nevertheless, it seems likely that multiple mechanisms may lead to 5-FU resistance. To the best of our knowledge, the Bel7402/5-FU cell line showed no EMT changes (29), 5-FU-resistant cell line associated with EMT is clearly required in HCC. Therefore, the HLF-Rs cells might be useful *in vitro* models for understanding 5-FU-resistant mechanisms in HCCs. Further, the HLF-R4 and HLF-R10 cells have different degrees of 5-FU resistance, so there are advantages to investigating the process of 5-FU resistance.

## Figures and Tables

**Table I:** Specific primers.

	Forward (5′-3′)	Reverse (5′-3′)
*RNR-R1*	AGAGAAGGAGAGGAACACAGCAG	AGCAAAGCCTTACCACCTCAAG
*RNR-R2*	GCCCCTGTTAAGTGGTGAAATC	GCCAGAATAAGACACTGGGTGAC
*MRP5*	TGAGACAGAAGCTCGATTCACC	AGGGAGGTTTTCTCGGTACCTC
*E-cadherin*	CTCTTCCAGGAACCTCTGTGATG	CCACACTGATGACTCCTGTGTTC
*Twist-1*	GCCGGAGACCTAGATGTCATTG	CTATCAGAATGCAGAGGTGTGAGG
*GAPDH*	GAGCCAAAAGGGTCATCATCTC	GGTCATGAGTCCTTCCACGATAC
